# Epidemiology of malignant tumors in patients with pemphigus: an analysis of trends from 1955 to 2021

**DOI:** 10.1007/s10238-024-01354-8

**Published:** 2024-05-17

**Authors:** Yue Luo, Xiaoya Fei, Mingxia Wang, Han Yang, Ying Zhang, Yiran Chen, Ying Luo, Xiaojie Ding, Chunjie Gao, Fang Shen, Ruiping Wang, Bin Li, Le Kuai, Qi Zheng, Miao Li, Jiankun Song

**Affiliations:** 1 Shanghai Skin Disease Hospital, Institute of Dermatology, School of Medicine, Tongji University, Shanghai, 200443 China; 2 Department of Dermatology, Yueyang Hospital of Integrated Traditional Chinese and Western Medicine, Shanghai University of Traditional Chinese Medicine, Shanghai, 200437 China; 3 Institute of Dermatology, Shanghai Academy of Traditional Chinese Medicine, Shanghai, 201203 China

**Keywords:** Pemphigus, Malignant tumors, Epidemiology

## Abstract

Background: The incidence of malignant tumors has increased in patients with non-paraneoplastic pemphigus, although there has been no systematic analysis of global epidemiology. Objective: To explore the epidemiology of various types of non-paraneoplastic pemphigus associated with malignant tumors. Methods: Five databases from establishment through October 20, 2023, were searched. STATA SE 17 was used for the data analysis. Subgroup, meta-regression, and sensitivity analyses were used to evaluate the heterogeneity of pooled studies. Results: A total of 6679 participants were included in our meta-analysis from 16 studies. The aggregated prevalence of tumors in patients diagnosed with pemphigus was 8%. The prevalence was 7% in patients with pemphigus vulgaris, 10% in those with pemphigus foliaceus, and 12% in individuals diagnosed with other types of pemphigus. The prevalence was 8% in Asia, 11% in Europe, and 8% in North America. From a country-specific perspective, patients with pemphigus from Israel, Greece, and Germany exhibited a higher prevalence of tumors at 11%. Furthermore, when categorized by the duration of the study period, the highest prevalence was observed in studies spanning 10 to 20 years, at 11%. Conclusion: These findings demonstrate the incidence and prevalence of malignant tumors in patients with non-paraneoplastic pemphigus, which may achieve early detection and intervention, and then reduce mortality rates.

**Supplementary Information:**

The online version contains supplementary material available at 10.1007/s10238-024-01354-8.

## Introduction

Pemphigus is a severe autoimmune skin disease characterized by chronic, recurrent blisters on the skin or mucous membranes. The primary pathogenesis is believed to be caused by the loss of intercellular adhesion due to autoantibodies targeting the desmoglein 1 and/or desmoglein 3, which are glycoproteins in the cadherin family that facilitate cell–cell adhesion in keratinocytes. It is typically classified into common, proliferative, deciduous, erythematous, and particular types. Its clinical features include the emergence of lax, thin-walled blisters, bullae, and erosions on the skin and mucosa. The overall estimated incidence was 7.2 per million inhabitants per year in Israel [1]. In Germany, it has been determined to be 1.5 per million per year [2]. Data from the 2002–2012 Nationwide Inpatient Sample showed the total annual inpatient cost-of-care for patients admitted with a primary diagnosis of pemphigus was $74,466,305 in America [3].

Systemic application of glucocorticoids is a first-line therapeutic drug. In addition, it is suggested that immunosuppressive agents should be used early in moderate and severe patients [4, 5]. The biological agent rituximab is usually used as a combination of systemic glucocorticoids and can also be used in combination with intravenous immunoglobulin (IVIG) [6]. Other therapies are plasma exchange, immune adsorption, and stem cell transplantation [7]. However, adverse reactions cannot be ignored. Long-term use of glucocorticoids and immunosuppressants can easily lead to infection, even leading to tumors. Patients with severe disease involving the skin and mucosa extensively across the body are susceptible to complications like hypoproteinemia and sepsis, which are life-threatening [8]. Paraneoplastic pemphigus (PNP), currently known to be associated with tumors, is a rare variant of pemphigus, accounting for roughly 5% of all cases. Almost all PNP patients have an association with tumors, benign or malignant [9]. Moreover, instances of other types of pemphigus occurring alongside malignant tumors should not be neglected.

However, there is currently no systematic epidemiological study on pemphigus combined with malignant tumors. Our purpose is to assess the epidemiology of various types of pemphigus associated with malignant tumors to achieve early detection and intervention and then reduce mortality rates.

## Methods

This study adhered to the PRISMA 2020 statement (Table S1) and the Meta-analysis of Observational Studies in Epidemiology (MOOSE) guidelines (Table S2).

### Search strategy

Two authors independently conducted a search of the PubMed, Embase, CNKI, Wanfang, and Sinomed databases from their establishment until October 20, 2023. The search strategy used both subject terms and free-word combinations. The primary search terms included “pemphigus,” “neoplasms,” “tumor,” “neoplasia,” “cancer,” and “malignancy.”

### Study selection

The criteria for inclusion in this study were as follows: (1) studies reporting the prevalence and/or incidence of comorbid tumors in pemphigus patients; (2) observational studies, including cohort, case–control, cross-sectional, and retrospective studies; (3) studies that had received ethics committee approval and had obtained signed informed consent from participants; (4) studies published in either Chinese or English; and (5) studies with no restrictions on sex, age, or country.

The exclusion criteria were: (1) duplicate studies; (2) non-observational studies; (3) studies with unavailable data; and (4) studies for which the full text was not accessible, making it impossible to ascertain whether they met the inclusion criteria.

### Data extraction

Two researchers independently extracted the following data: first author’s name, year of publication, period of study, study type, region (country), types of pemphigus, types of tumors, number of pemphigus cases with co-occurring tumors, and prevalence and/or incidence of comorbidities.

### Quality assessment

Two authors independently evaluated the pooled studies. For cross-sectional studies, the Agency for Healthcare Research and Quality (AHRQ) tool was employed to assess the risk of bias. For case–control and cohort studies, the Newcastle–Ottawa Scale (NOS) was used for evaluation. Each study was assigned a specific score on the following scale: a score of 0–3 was defined as low quality, 4–7 as medium quality, and 8–11 as high quality.

### Statistical analysis

Data analysis was performed using STATA SE 17. Heterogeneity was assessed using the *I*^2^ test. If the *I*^2^ value was less than 50%, a fixed-effects model was applied; if not, a random-effects model was utilized. Heterogeneity was further addressed through subgroup, meta-regression, and sensitivity analyses. To determine the presence of publication bias, both Egger’s and Begg’s linear regression tests were employed. If publication bias was detected, the trim-and-fill method was enacted to estimate the number of potentially missing studies and correct for the bias.

## Results

### Characteristics of the included studies

**Fig. 1:**
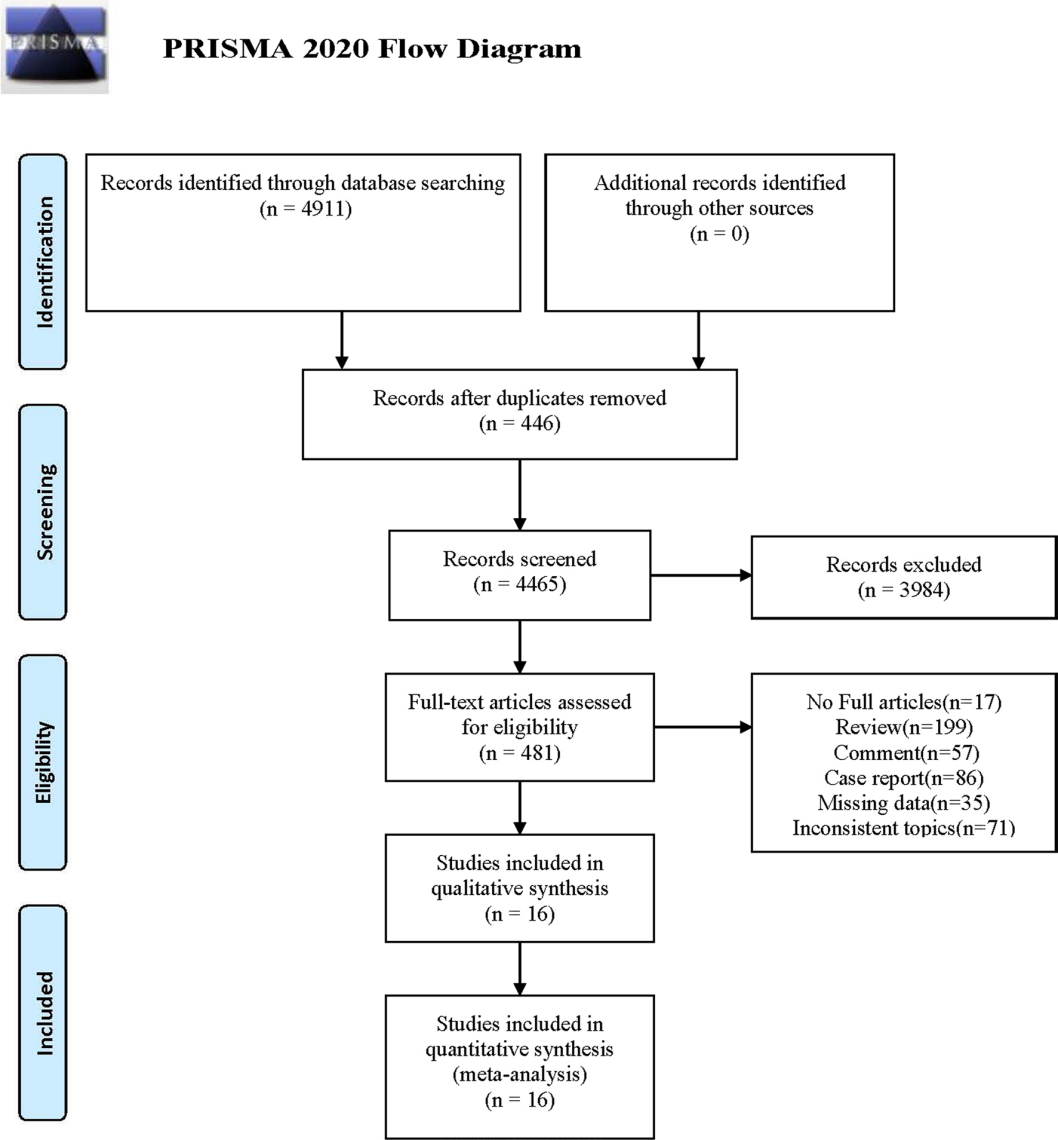
Flow diagram according to PRISMA 2020 guidelines

The initial literature search yielded 4,911 studies. After removing duplicates, the titles and abstracts of the remaining 4465 studies were reviewed for relevance. This resulted in 481 studies being chosen for a more detailed, full-text evaluation. However, 465 of these studies were subsequently excluded due to the lack of clear diagnostic criteria, unspecified research methodology, incomplete data, or mismatch with the study topic. In the end, 16 studies (6679 participants) satisfied the inclusion criteria and were included in this meta-analysis (Fig. 1 and Table 1).

**Table 1:** Characteristics of the included studies

Author/publish year	Study year	Study type	Region	Country	Generation	Type of pemphigus	Pemphigus (n)	Tumor in Pemphigus(N)
Krain et al., 1974	1955–1973	Retrospective study	North America	USA	Adult	Pemphigus vulgaris	51	8
Morioka et al., 1994	1981–1986	Cohort study	Asia	Japan	Adult	Pemphigus vulgaris	279	11
						Pemphigus foliaceus	195	12
						Other pemphigus	22	2
Kyriakis et al., 1998	1987–1989	Cohort study	Europe	Greece	UN	Pemphigus vulgaris	37	4
Tsankov et al., 2000	1980–1995	Retrospective study	Europe	Bulgaria	Adult	Pemphigus vulgaris	57	3
						Other pemphigus	2	1
Goon et al., 2001	1995–1997	Retrospective study	Asia	Singapore	Adult	Pemphigus vulgaris	31	1
						Pemphigus foliaceus	16	1
Zeng et al., 2002	1990–2001	Cohort study	Asia	Saudi Arabia	Adult	UN	92	6
Iwashita et al., 2007	1975–2006	Retrospective study	Asia	Japan	Adult	Pemphigus vulgaris	50	3
						Pemphigus foliaceus	37	6
Heelan et al., 2015	2012–2013	Cross-sectional study	North America	Canada	Adult	UN	295	23
Schulze et al., 2015	2008–2011	Retrospective study	Europe	Germany	Adult	Pemphigus vulgaris	860	93
						Pemphigus foliaceus	103	17
Toosi et al., 2016	1993-2013	Retrospective study	North America	USA	Adult	Other pemphigus	13	2
Akarsu et al., 2017	2011-2015	Cohort study	Asia	Turkey	Adult	Pemphigus vulgaris	26	1
Kridin et al., 2018 (a)	1990-2011	Cohort study	Asia	Israel	Adult	Pemphigus vulgaris	184	15
Kridin et al., 2018 (b)	2004-2014	Cross-sectional study	Asia	Israel	Adult	UN	1985	258
Kridin et al., 2018 (c)	2017	Cross-sectional study	North America	USA	Adult	UN	1985	93
Jelti et al., 2019	2004-2013	Retrospective study	Europe	France	Adult	Pemphigus vulgaris	155	15
						Pemphigus foliaceus	67	5
Zou H., 2021	2018-2021	Retrospective study	Asia	China	Adult	UN	104	8

*n* number, *UN* Unknown

Legends: Other pemphigus include vegetans pemphigus (Morioka et al., 1994), indeterminates pemphigus (Morioka et al., 1994), superficial pemphigus (Tsankov et al., 2000), herpetiformis pemphigus (Tsankov et al., 2000), IgG/IgA pemphigus (Toosi et al., 2016)

### Study quality

There was no suitable evaluation instrument for the retrospective studies, so these were excluded from the quality assessments [10–17]. Two studies were classified as high quality [18, 19], while the remaining studies [20–25] were given a medium-quality rating. The details of the quality evaluation can be found in Supplementary Tables S3 and S4.

### Outcomes

#### Prevalence

**Fig. 2:**
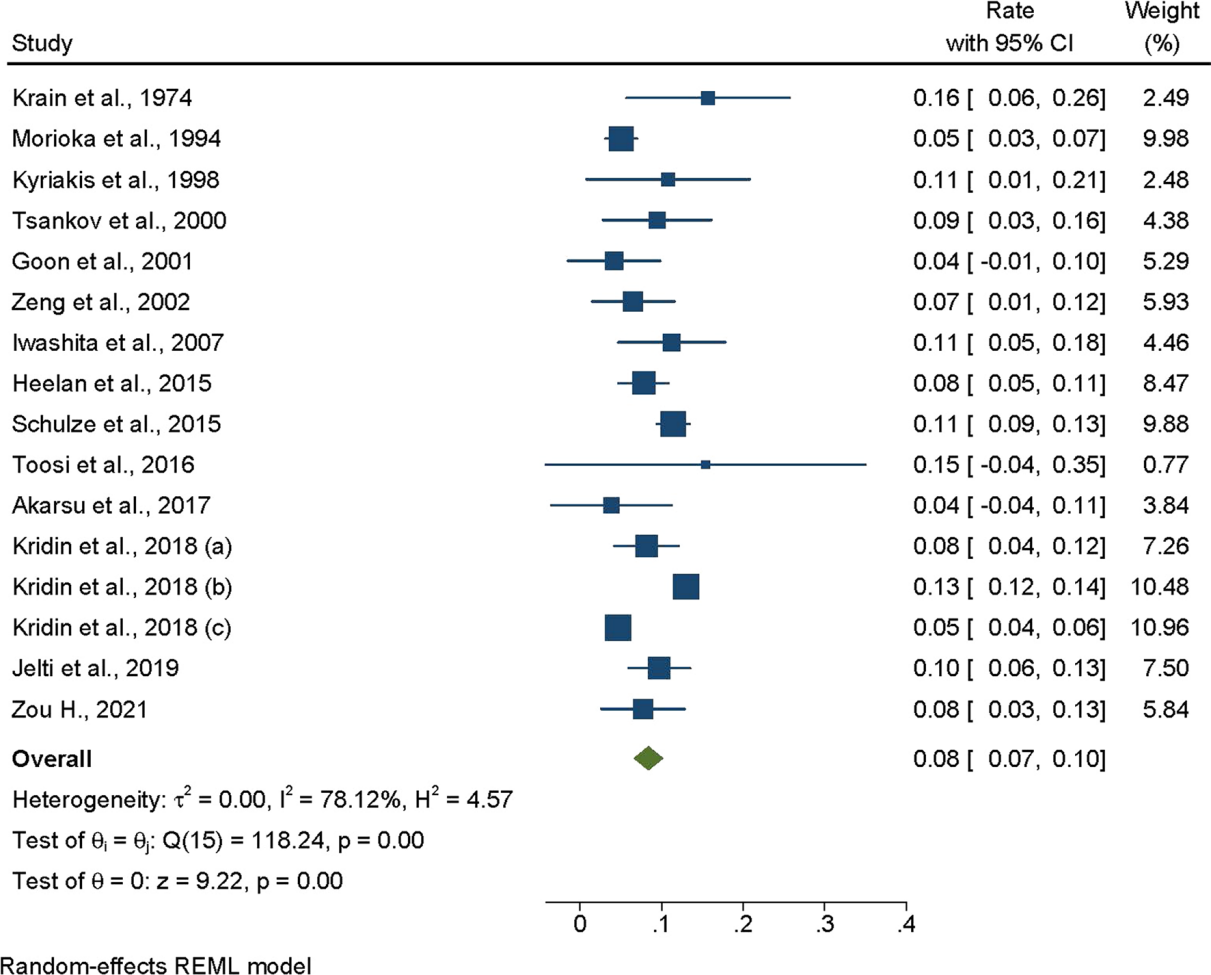
Overall prevalence of malignant tumors in non-paraneoplastic pemphigus

The overall prevalence of tumors in patients diagnosed with pemphigus was found to be 8% (95% CI: 0.07, 0.10) (Fig. 2). A subgroup analysis was conducted, segregated by different types of pemphigus. The prevalence in patients with pemphigus vulgaris was 7% (95% CI: 0.05, 0.10), compared to 10% (95% CI: 0.05, 0.14) in those with pemphigus foliaceus, and 12% (95% CI: 0.02, 0.22) in individuals diagnosed with other types of pemphigus (Figure S1).

The prevalence of malignancy in patients with pemphigus was reported solely by studies conducted in Asia, Europe, and North America. In terms of continental distribution, the prevalence was 8% (95% CI: 0.05, 0.10) in Asia, 11% (95% CI: 0.09, 0.13) in Europe, and 8% (95% CI: 0.03, 0.12) in North America (Figure S2). Within Asia, six countries reported the following prevalence rates: China at 8% (95% CI: 0.03, 0.13); Japan at 7% (95% CI: 0.01, 0.13); Singapore at 4% (95% CI: − 0.01, 0.10); Turkey at 4% (95% CI: − 0.04, 0.11); Israel at 11% (95% CI: 0.06, 0.16); and Saudi Arabia at 7% (95% CI: 0.01, 0.12). In Europe, four countries reported these figures: Greece at 11% (95% CI: 0.01, 0.21); Bulgaria at 9% (95% CI: 0.03, 0.16); Germany at 11% (95% CI: 0.09, 0.13); and France at 10% (95% CI: 0.06, 0.13). In North America, Canada, and the United States of America (USA) reported comorbid malignancy and pemphigus prevalences of 8% (95% CI: 0.05, 0.11) and 10% (95% CI: 0.01, 0.18), respectively (Figure S3).

Furthermore, the prevalence varied based on the type and duration of the study. In cohort studies, the prevalence was recorded at 6% (95% CI: 0.04, 0.08); in cross-sectional studies, it was 8% (95% CI: 0.04, 0.13); and in retrospective studies, it was 10% (95% CI: 0.08, 0.12) (Figure S4). Additionally, when sorted by the duration of the study period, the prevalence in studies lasting fewer than 10 years was 7% (95% CI: 0.05, 0.09); in studies spanning 10 to 20 years, it was 11% (95% CI: 0.08, 0.14); and in those that extended beyond 20 years, it was 9% (95% CI: 0.06, 0.12) (Figure S5).

#### Heterogeneity analysis

**Fig. 3:**
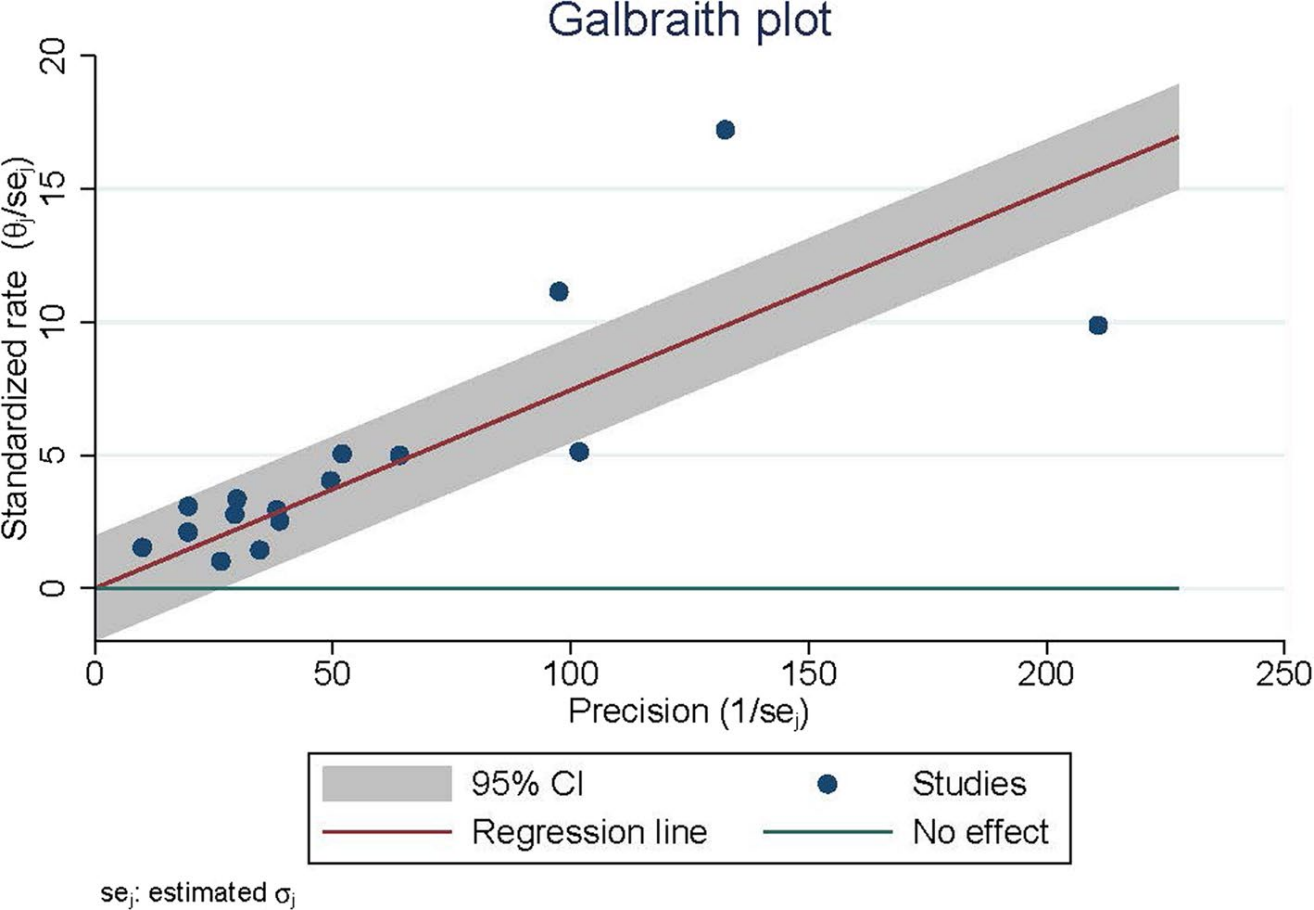
Galbraith plot

The Galbraith plot shows that some studies fall outside the confidence interval boundaries, and the steep gradient of the scatter plot indicates existing heterogeneity (Fig. 3).

A meta-regression analysis was performed to identify potential sources of this heterogeneity in prevalence. Unexpectedly, factors such as study design, study quality, geographical region, study duration, and publication year were not identified as sources of heterogeneity in the prevalence of malignancy among patients with pemphigus (Table S5). Nevertheless, subgroup analyses were conducted to address the observed heterogeneity.

#### Sensitivity analyses

**Fig. 4:**
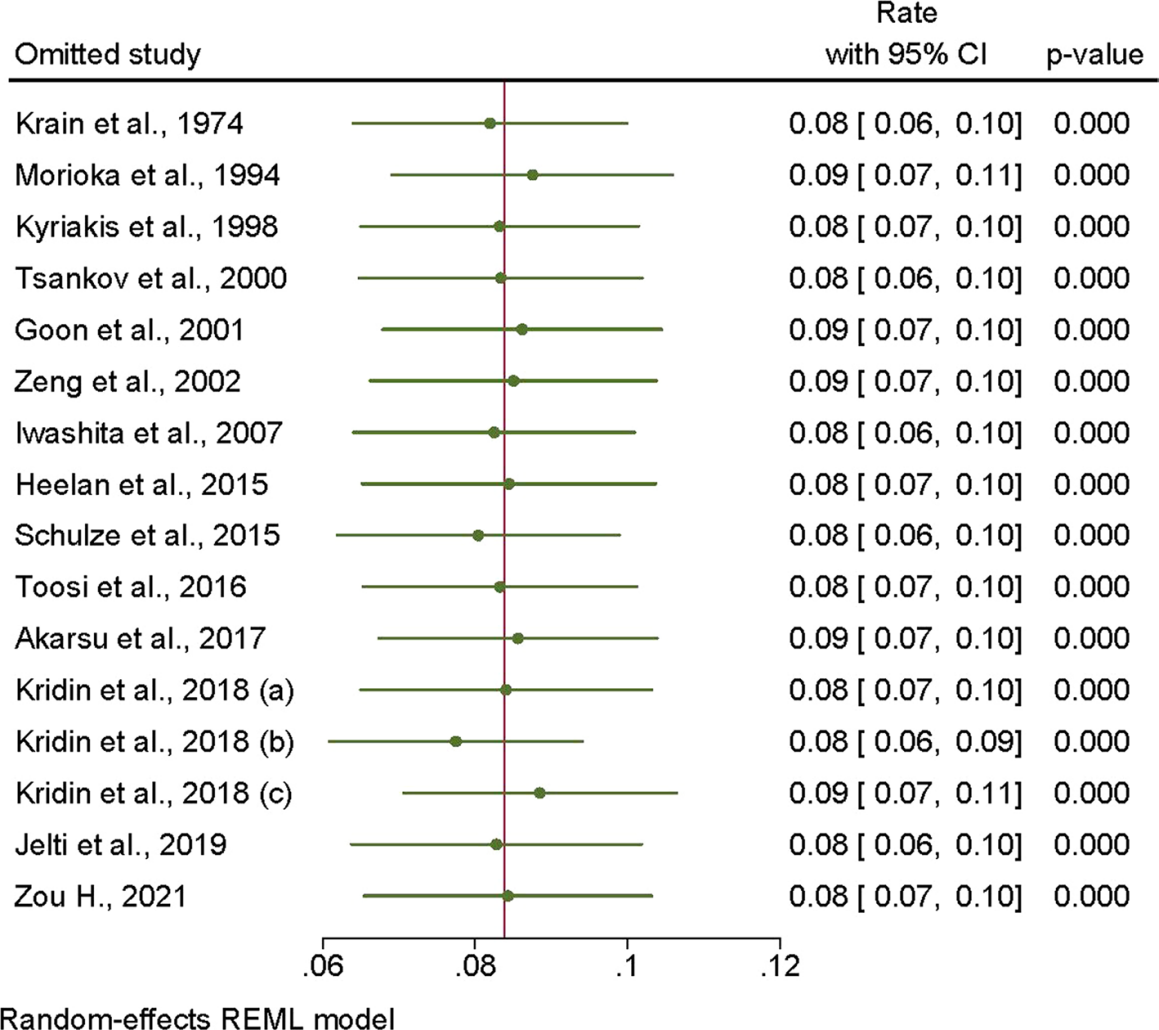
The leave-one-out meta-analysis

Separate sensitivity analyses were performed to investigate the prevalence of malignancy in patients with pemphigus. The leave-one-out meta-analysis indicates that the findings are both stable and reliable (Fig. 4).

### Publication bias

By employing Egger’s and Begg’s linear regression tests, we assessed the potential for publication bias concerning the prevalence of malignancy in patients with pemphigus. Both linear regression tests revealed no evidence of publication bias in relation to malignancy prevalence among patients with pemphigus (Egger test, *P* = 0.4905; Begg’s test, *P* = 0.6204).

## Discussion

This study scrutinized the global epidemiology of pemphigus and tumors. Overall, the aggregated prevalence of tumors in patients diagnosed with pemphigus was 8%. The prevalence was 7% in patients with pemphigus vulgaris, 10% in those with pemphigus foliaceus, and 12% in individuals diagnosed with other types of pemphigus. The prevalence was 8% in Asia, 11% in Europe, and 8% in North America. From a country-specific perspective, patients with pemphigus from Israel, Greece, and Germany exhibited a higher prevalence of tumors at 11%. Furthermore, when categorized by the duration of the study period, the highest prevalence was observed in studies spanning 10 to 20 years, at 11%. Therefore, it is necessary to strengthen the screening of tumors in patients with pemphigus, especially for those with a disease course of more than 10 years.

The guideline for diagnosis and treatment–guided of pemphigus formulated by the European Dermatology Forum (EDF) in cooperation with the European Academy of Dermatology and Venereology (EADV) have pointed out concerns about pemphigus combined with malignant tumors [4]. For instance, a retrospective study indicated that six out of 96 patients with pemphigus were also diagnosed with malignant tumors, which histopathological and immunopathological evaluations did not reveal the typical traits of paraneoplastic pemphigus [25]. A population-based study between 1985 pemphigus cases and 9847 control individuals from 2004 to 2012 found that the incidence of pemphigus co-occurring with esophageal (OR = 2.9, 95% CI 1.1, 7.4) and laryngeal cancer (OR = 2.0, 95% CI 1.0, 4.1) was significantly higher than in the control group [21]. In a British population-based study using a computerized database, mortality among Pemphigus vulgaris patients was 3.3 times higher than among age- and sex matched control subjects [26]. The medical community and public have acknowledged that malignancy impacts the survival rate in patients with pemphigus [27]. Therefore, early detection, diagnosis, and treatment of potential malignant tumors for patients with pemphigus are of great significance. Our study found that the incidence rate of pemphigus vulgaris and pemphigus foliaceus with malignant tumors was higher in all types, of which the probability of malignant tumors occurring in pemphigus foliaceus was higher than that in pemphigus vulgaris. In a recent German case–control study, PV was found to be associated with oropharyngeal, gastrointestinal, colon neoplasms and hematologic malignancies, whereas PF was associated with nonmelanoma skin cancer [14]. A recent population-based study demonstrated that the prevalence of chronic leukemia, multiple myeloma, and non-Hodgkin lymphoma was greater in patients with pemphigus than in controls [22]. In addition, we found that Israel, Greece, and Germany patients with pemphigus presented a higher prevalence of tumors at 11% (Figure S3). This may be different from the incidence rate of various forms of pemphigus in different countries. Pemphigus vulgaris is the most common in Europe and the USA and has been recognized as the most prevalent type of pemphigus, comprising up to 70% of all cases of pemphigus [28].

There were currently no similar systematic comments published. Our study has some innovative points in several respects. First, we are the only study to date to analyze the epidemiology of malignancy in patients with different types of pemphigus. Second, we systematically analyzed malignancy epidemiology in patients with different types of pemphigus in different countries and regions worldwide. Attention to the incidence of malignant tumors in patients with different types of pemphigus in different regions. For limitation, the comorbidity rates of these two diseases have not been reported in some countries, and larger sample size reports are needed to support our conclusion. However, when we conduct sensitivity analysis, the results are relatively stable.

## Conclusion

This is the only study to date to analyze the epidemiology of malignancy in patients with different types of pemphigus. Our findings demonstrate the incidence and prevalence of malignant tumors in patients with non-paraneoplastic pemphigus, which may achieve early detection and intervention, and then reduce mortality rates.

## Supplementary Information

Below is the link to the electronic supplementary material.

Supplementary file1 (DOCX 1251 KB)

## Declarations

### Data availability Statement

All data generated in this study are included in this article and its supplementary information files.
