# Supplementary material for: Epidemiology of malignant tumors in patients with pemphigus: an analysis of trends from 1955 to 2021
Source: Clin Exp Med. 2024 May 17;24(1):100. doi: 10.1007/s10238-024-01354-8 (PMC11101525; doi:10.1007/s10238-024-01354-8)
Supplement: Supplementary file 1 — Supplementary file1 (DOCX 1251 KB) [file 10238_2024_1354_MOESM1_ESM.docx]

**Supplementary materials**

**Epidemiology of malignant tumors in patients with** **pemphigus: An analysis of trends from 1955 to 2021**

Yue Luo^1,*^, Xiaoya Fei^1,*^, Mingxia Wang^1,*^, Han Yang^2^, Ying Zhang^1^, Yiran Chen^2^, Ying Luo^3^, Xiaojie Ding^2^, Chunjie Gao^1^, Fang Shen^1^, Ruiping Wang^1^, Bin Li^1, 3^, Le Kuai^2^, Qi Zheng^1,#^, Miao li^2,#^, Jiankun Song^1,#^

**Figure S1. Prevalence of malignant tumors in non-paraneoplastic pemphigus by type.**


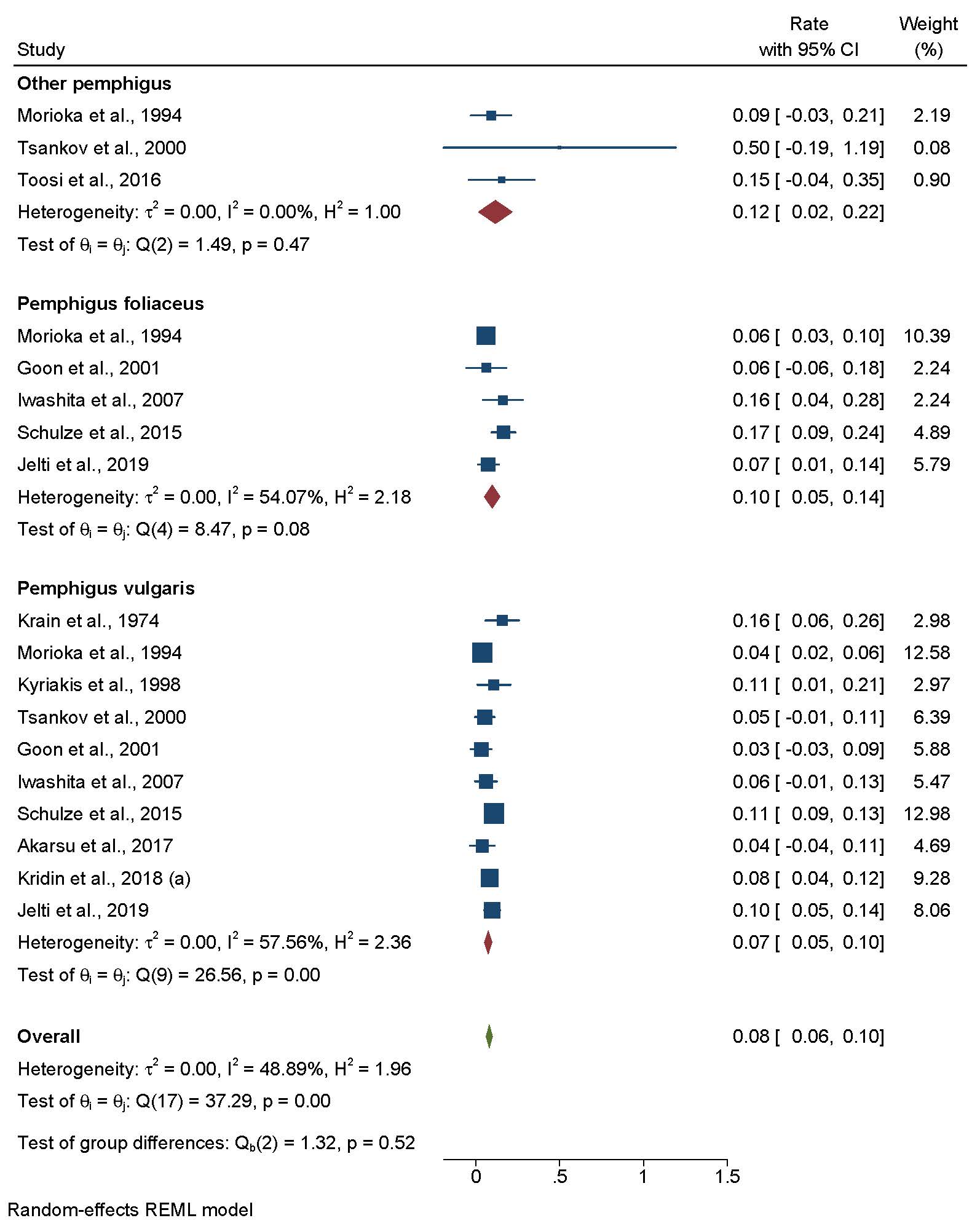


**Figure S2. Prevalence of malignant tumors in non-paraneoplastic pemphigus by region.**


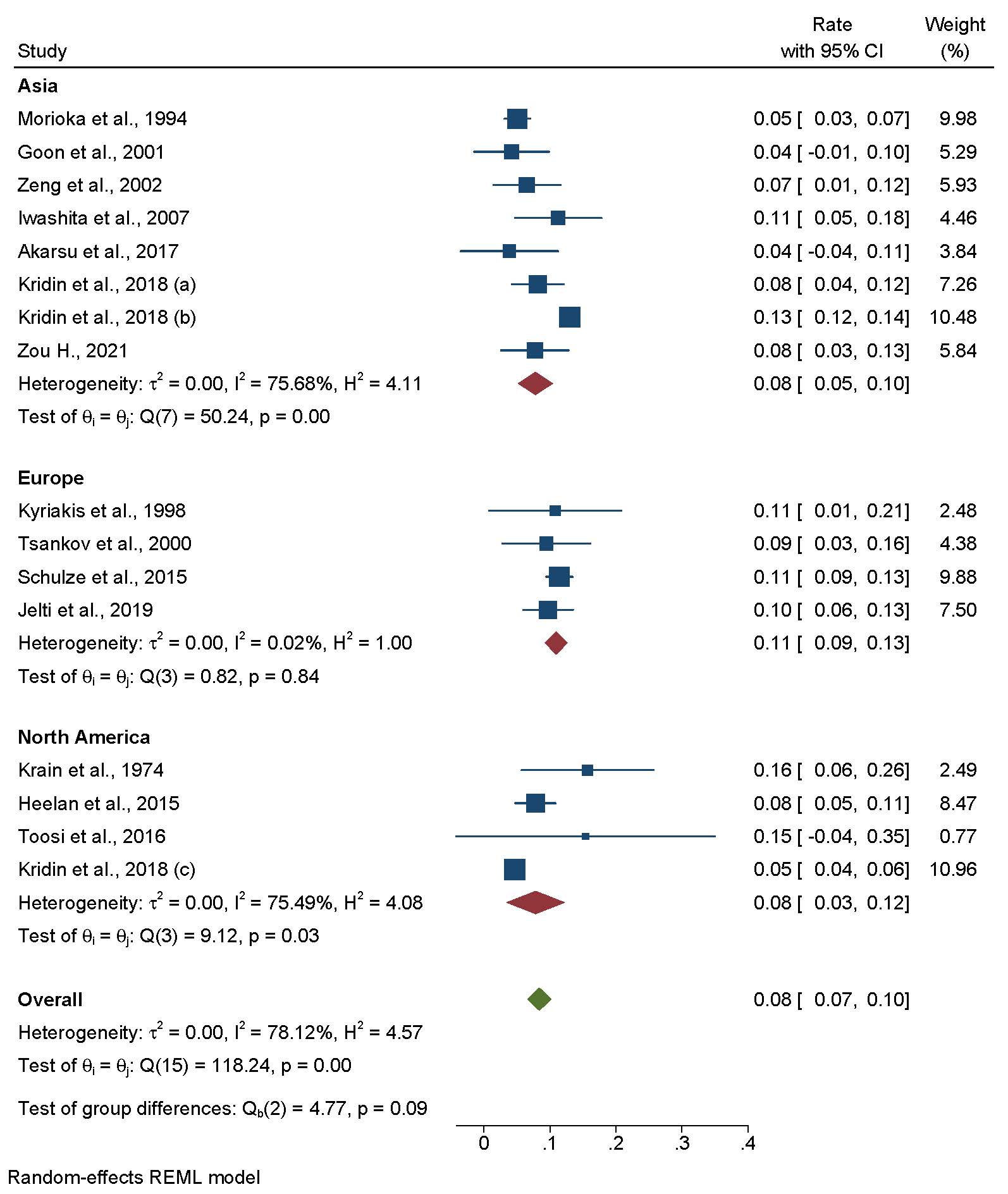


**Figure S3. Prevalence of malignant tumors in non-paraneoplastic pemphigus by country.**


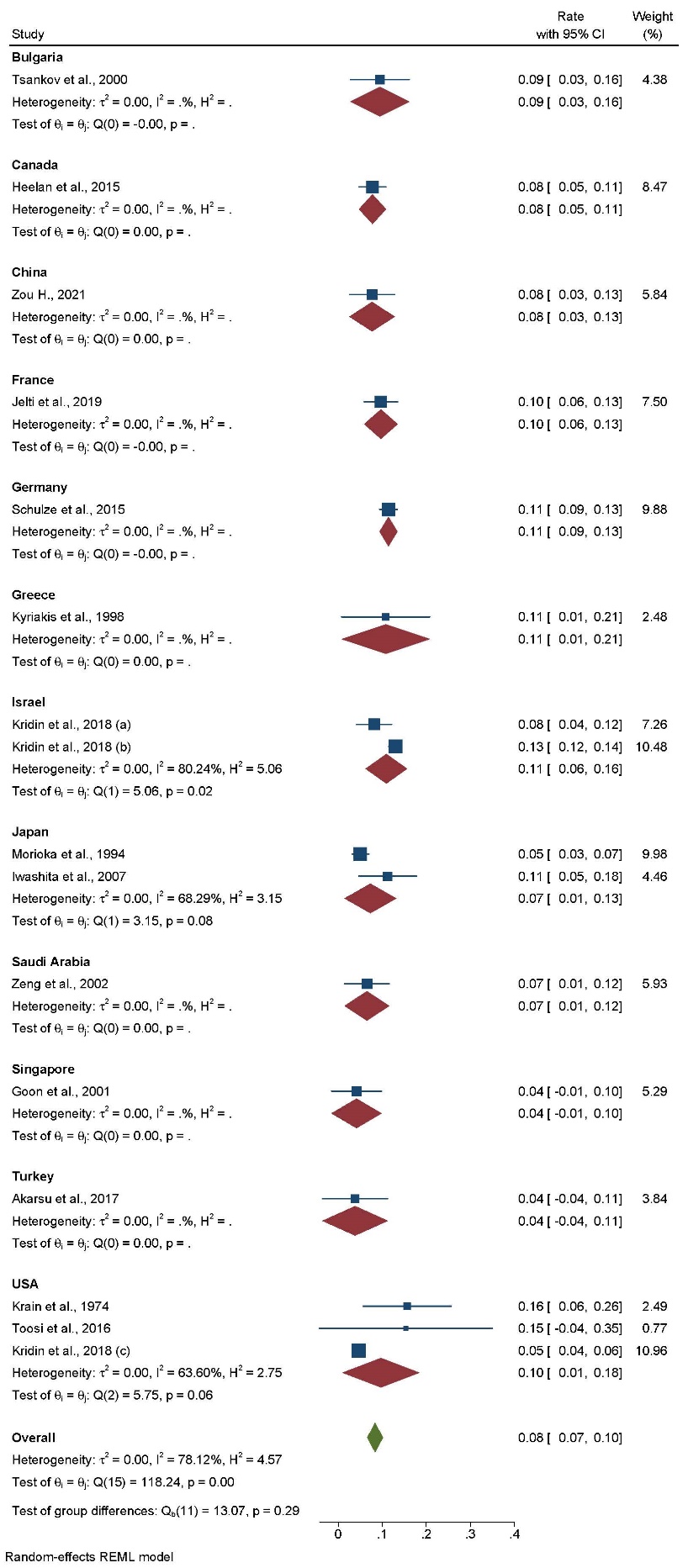


**Figure S4. Prevalence of malignant tumors in non-paraneoplastic pemphigus by design.**


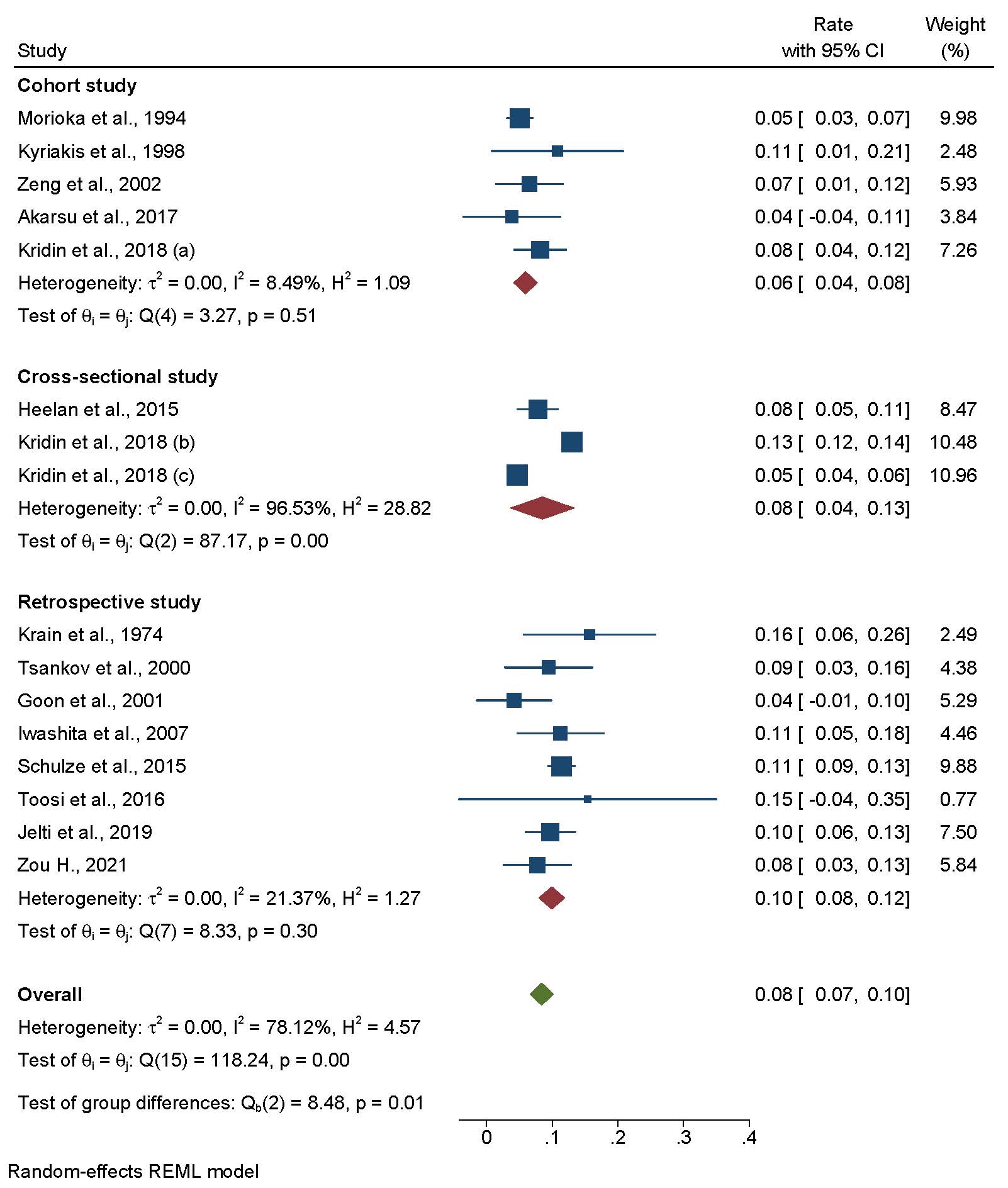


**Figure S5. Prevalence of malignant tumors in non-paraneoplastic pemphigus by course group.**


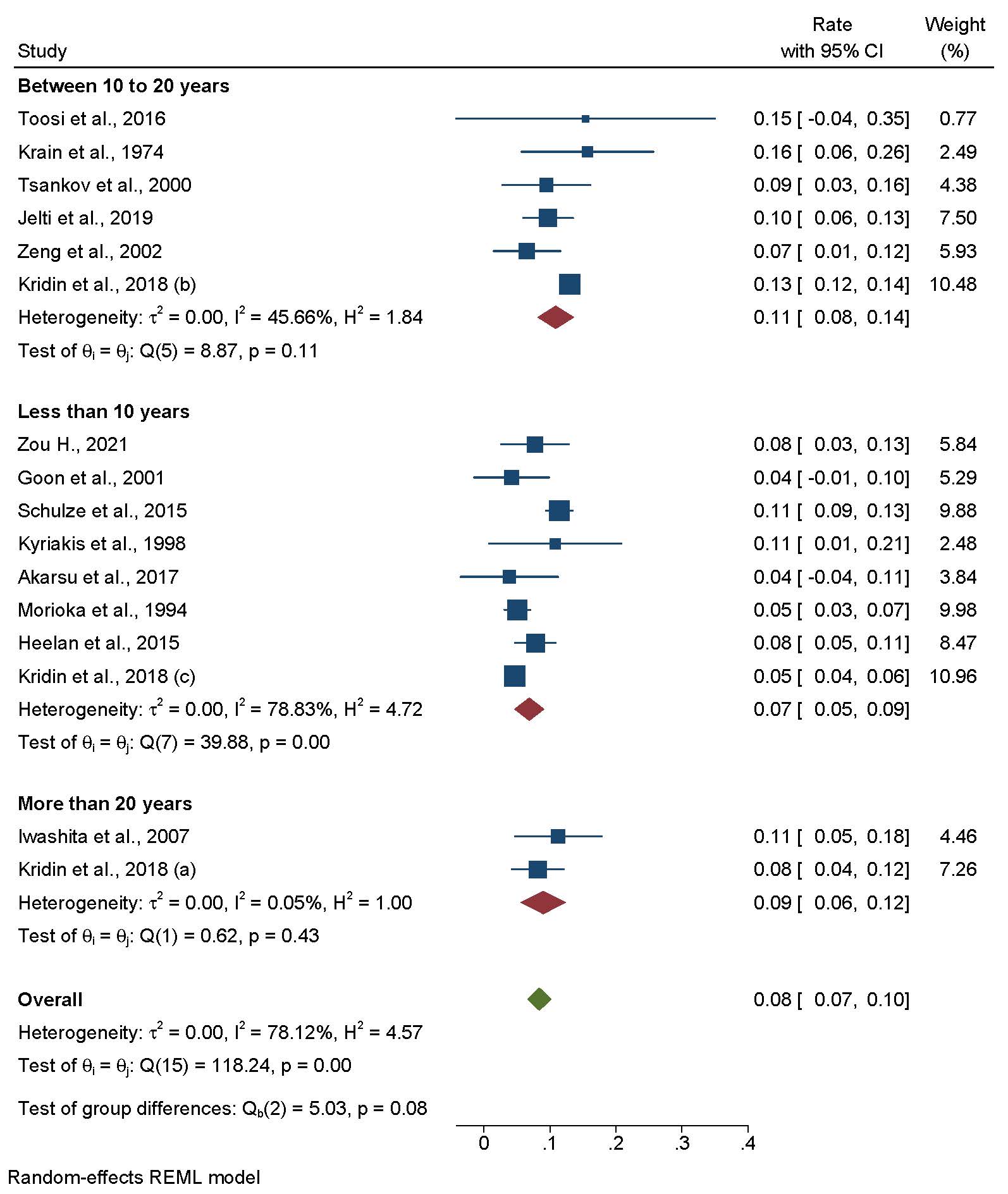


**Table S1. Preferred Reporting Items for Systematic Reviews and Meta-Analyses (PRISMA) checklist 2020.**

| **Section and Topic** | **Item #** | **Checklist item** | **Location where item is reported** |
| --- | --- | --- | --- |
| **TITLE** | | |  |
| Title | 1 | Identify the report as a systematic review. | **Page 1 (Title)** |
| **ABSTRACT** | | |  |
| Abstract | 2 | See the PRISMA 2020 for Abstracts checklist. | **Pages 2-3 (Abstract)** |
| **INTRODUCTION** | | |  |
| Rationale | 3 | Describe the rationale for the review in the context of existing knowledge. | **Pages 3-5 (Introduction)** |
| Objectives | 4 | Provide an explicit statement of the objective(s) or question(s) the review addresses. | **Pages 3-5 (Introduction)** |
| **METHODS** | | |  |
| Eligibility criteria | 5 | Specify the inclusion and exclusion criteria for the review and how studies were grouped for the syntheses. | **Page 6 (Search strategy)** |
| Information sources | 6 | Specify all databases, registers, websites, organisations, reference lists and other sources searched or consulted to identify studies. Specify the date when each source was last searched or consulted. | **Page 6 (Search strategy)** |
| Search strategy | 7 | Present the full search strategies for all databases, registers and websites, including any filters and limits used. | **Page 6 (Search strategy)** |
| Selection process | 8 | Specify the methods used to decide whether a study met the inclusion criteria of the review, including how many reviewers screened each record and each report retrieved, whether they worked independently, and if applicable, details of automation tools used in the process. | **Page 6-7 (Inclusion and exclusion criteria)** |
| Data collection process | 9 | Specify the methods used to collect data from reports, including how many reviewers collected data from each report, whether they worked independently, any processes for obtaining or confirming data from study investigators, and if applicable, details of automation tools used in the process. | **Page 7 (Data extraction)** |
| Data items | 10a | List and define all outcomes for which data were sought. Specify whether all results that were compatible with each outcome domain in each study were sought (e.g. for all measures, time points, analyses), and if not, the methods used to decide which results to collect. | **Page 7 (Data extraction)** |
|  | 10b | List and define all other variables for which data were sought (e.g. participant and intervention characteristics, funding sources). Describe any assumptions made about any missing or unclear information. | **Page 7 (Data extraction)** |
| Study risk of bias assessment | 11 | Specify the methods used to assess risk of bias in the included studies, including details of the tool(s) used, how many reviewers assessed each study and whether they worked independently, and if applicable, details of automation tools used in the process. | **Page 7 (Risk of bias in individual studies)** |
| Effect measures | 12 | Specify for each outcome the effect measure(s) (e.g. risk ratio, mean difference) used in the synthesis or presentation of results. | **Page 8 (Statistical analysis)** |
| Synthesis methods | 13a | Describe the processes used to decide which studies were eligible for each synthesis (e.g. tabulating the study intervention characteristics and comparing against the planned groups for each synthesis (item #5)). | **Page 8 (Statistical analysis)** |
|  | 13b | Describe any methods required to prepare the data for presentation or synthesis, such as handling of missing summary statistics, or data conversions. | **Page 8 (Statistical analysis)** |
|  | 13c | Describe any methods used to tabulate or visually display results of individual studies and syntheses. | **Page 8(Statistical analysis)** |
|  | 13d | Describe any methods used to synthesize results and provide a rationale for the choice(s). If meta-analysis was performed, describe the model(s), method(s) to identify the presence and extent of statistical heterogeneity, and software package(s) used. | **Page 8 (Statistical analysis)** |
|  | 13e | Describe any methods used to explore possible causes of heterogeneity among study results (e.g. subgroup analysis, meta-regression). | **Page 8 (Statistical analysis)** |
|  | 13f | Describe any sensitivity analyses conducted to assess robustness of the synthesized results. | **Page 8 (Statistical analysis)** |
| Reporting bias assessment | 14 | Describe any methods used to assess risk of bias due to missing results in a synthesis (arising from reporting biases). | **Page 7 (Risk of bias in individual studies)** |
| Certainty assessment | 15 | Describe any methods used to assess certainty (or confidence) in the body of evidence for an outcome. | **Page 8 (Statistical analysis)** |
| **RESULTS** | | |  |
| Study selection | 16a | Describe the results of the search and selection process, from the number of records identified in the search to the number of studies included in the review, ideally using a flow diagram. | **Pages 8 (Study selection of included studies)** |
|  | 16b | Cite studies that might appear to meet the inclusion criteria, but which were excluded, and explain why they were excluded. | **Pages 8 (Study selection of included studies)** |
| Study characteristics | 17 | Cite each included study and present its characteristics. | **Pages 8 (study characteristics)** |
| Risk of bias in studies | 18 | Present assessments of risk of bias for each included study. | **Pages 8-9 (Risk of bias assessments)** |
| Results of individual studies | 19 | For all outcomes, present, for each study: (a) summary statistics for each group (where appropriate) and (b) an effect estimate and its precision (e.g. confidence/credible interval), ideally using structured tables or plots. | **Pages 9-11 (Primary outcomes / Secondary outcomes)** |
| Results of syntheses | 20a | For each synthesis, briefly summarise the characteristics and risk of bias among contributing studies. | **Pages 9-11 (Primary outcomes / Secondary outcomes)** |
|  | 20b | Present results of all statistical syntheses conducted. If meta-analysis was done, present for each the summary estimate and its precision (e.g. confidence/credible interval) and measures of statistical heterogeneity. If comparing groups, describe the direction of the effect. | **Pages 9-11 (Primary outcomes / Secondary outcomes)** |
|  | 20c | Present results of all investigations of possible causes of heterogeneity among study results. | **Pages 9-11 (Primary outcomes / Secondary outcomes)** |
|  | 20d | Present results of all sensitivity analyses conducted to assess the robustness of the synthesized results. | **Pages 9-11 (Primary outcomes / Secondary outcomes)** |
| Reporting biases | 21 | Present assessments of risk of bias due to missing results (arising from reporting biases) for each synthesis assessed. | **Pages 9 (Risk of bias assessments)** |
| Certainty of evidence | 22 | Present assessments of certainty (or confidence) in the body of evidence for each outcome assessed. | **Pages 9-11 (Primary outcomes / Secondary outcomes)** |
| **DISCUSSION** | | |  |
| Discussion | 23a | Provide a general interpretation of the results in the context of other evidence. | **Pages 11-15 (Discussion)** |
|  | 23b | Discuss any limitations of the evidence included in the review. | **Pages** **15 (Limitations)** |
|  | 23c | Discuss any limitations of the review processes used. | **Pages 15 (Limitations)** |
|  | 23d | Discuss implications of the results for practice, policy, and future research. | **Pages 11-15(Discussion)** |
| **OTHER INFORMATION** | | |  |
| Registration and protocol | 24a | Provide registration information for the review, including register name and registration number, or state that the review was not registered. | **Page 6 (Methods)** |
|  | 24b | Indicate where the review protocol can be accessed, or state that a protocol was not prepared. | **Page 6 (Methods)** |
|  | 24c | Describe and explain any amendments to information provided at registration or in the protocol. | **Page 6 (Methods)** |
| Support | 25 | Describe sources of financial or non-financial support for the review, and the role of the funders or sponsors in the review. | **Page 16 (Funding)** |
| Competing interests | 26 | Declare any competing interests of review authors. | **Page 16 (Declaration of interest)** |
| Availability of data, code and other materials | 27 | Report which of the following are publicly available and where they can be found: template data collection forms; data extracted from included studies; data used for all analyses; analytic code; any other materials used in the review. | **Page 16 (Data Availability Statement)** |

*From:*  Page MJ, McKenzie JE, Bossuyt PM, Boutron I, Hoffmann TC, Mulrow CD, et al. The PRISMA 2020 statement: an updated guideline for reporting systematic reviews. BMJ 2021;372:n71. doi: 10.1136/bmj.n71

**Table S2 MOOSE Checklist**

**MOOSE (Meta-analyses Of Observational Studies in Epidemiology) Checklist**

A reporting checklist for Authors, Editors, and Reviewers of Meta-analyses of Observational Studies. You must report the page number in your manuscript where you consider each of the items listed in this checklist. If you have not included this information, either revise your manuscript accordingly before submitting or note N/A.

| **Reporting Criteria** | **Reported (Yes/No)** | | | **Reported on Page No.** | | |
| --- | --- | --- | --- | --- | --- | --- |
| **Reporting of Background** |  | | |  | | |
| Problem definition |  | Yes |  |  | 3 |  |
| Hypothesis statement |  | No |  |  |  |  |
| Description of Study Outcome(s) |  | No |  |  |  |  |
| Type of exposure or intervention used |  | Yes |  |  | 4-5 |  |
| Type of study design used |  | Yes |  |  | 4-5 |  |
| Study population |  | Yes |  |  | 4-5 |  |
| **Reporting of Search Strategy** |  | | |  | | |
| Qualifications of searchers (eg, librarians  and investigators) | No | | |  | | |
| Search strategy, including time period  included in the synthesis and keywords | Yes | | | 6 | | |
| Effort to include all available studies,  including contact with authors | No | | |  | | |
|  |  |  |  |  |  |  |
| Databases and registries searched |  | Yes |  |  | 6 |  |
| Search software used, name and version, including special features used  (eg, explosion) | No | | |  | | |
| Use of hand searching (eg, reference  lists of obtained articles) | No | | |  | | |
| List of citations located and those  excluded, including justification | Yes | | | 6-7 | | |
| Method for addressing articles  published in languages other than English | Yes | | | 6 | | |
| Method of handling abstracts and  unpublished studies | Yes | | | 6 | | |
| Description of any contact with authors |  | No |  |  |  |  |
| **Reporting of Methods** |  | | |  | | |
| Description of relevance or appropriateness of studies assembled for  assessing the hypothesis to be tested | No | | |  | | |
| Rationale for the selection and coding of data (eg, sound clinical principles or  convenience) | No | | |  | | |
| Documentation of how data were classified and coded (eg, multiple raters,  blinding, and interrater reliability) | N0 | | |  | | |
| Assessment of confounding (eg, comparability of cases and controls in  studies where appropriate | No | | |  | | |

| **Reporting Criteria** | **Reported (Yes/No)** | | | **Reported on Page No.** | | |
| --- | --- | --- | --- | --- | --- | --- |
| Assessment of study quality, including blinding of quality assessors; stratification or regression on possible  predictors of study results | No | | |  | | |
| Assessment of heterogeneity |  | Yes |  |  | 6-8 |  |
| Description of statistical methods (eg, complete description of fixed or random effects models, justification of whether the chosen models account for predictors of study results, dose-response models, or cumulative meta-analysis) in sufficient  detail to be replicated | Yes | | | 6-8 | | |
| Provision of appropriate tables and  graphics | Yes | | | 6-8 | | |
| **Reporting of Results** |  | | |  | | |
| Table giving descriptive information for  each study included | Yes | | | 8 | | |
| Results of sensitivity testing (eg,  subgroup analysis) | Yes | | | 8-9 | | |
| Indication of statistical uncertainty of  findings | No | | |  | | |
| **Reporting of Discussion** |  | | |  | | |
| Quantitative assessment of bias (eg,  publication bias) | Yes | | | 7 | | |
| Justification for exclusion (eg, exclusion  of non–English-language citations) | Yes | | | 6-7 | | |
| Assessment of quality of included studies |  | Yes |  |  | 7 |  |
| **Reporting of Conclusions** |  | | |  | | |
| Consideration of alternative explanations  for observed results | No | | |  | | |
| Generalization of the conclusions (ie, appropriate for the data presented and  within the domain of the literature review) | Yes | | | 15 | | |
| Guidelines for future research |  | Yes |  |  | 14-15 |  |
| Disclosure of funding source |  | Yes |  |  | 16 |  |

**Once you have completed this checklist, please save a copy and upload it as part of your submission. DO NOT include this checklist as part of the main manuscript document. It must be uploaded as a separate file.**

| **Table S3. Risk-of-bias of cross-sectional studies assessed using the Agency for Healthcare Research and Quality tool.** | | | | | | | | | | | | |
| --- | --- | --- | --- | --- | --- | --- | --- | --- | --- | --- | --- | --- |
| ***Studies*** | ***Q1*** | ***Q2*** | ***Q3*** | ***Q4*** | ***Q5*** | ***Q6*** | ***Q7*** | ***Q8*** | ***Q9*** | ***Q10*** | ***Q11*** | ***Scores*** |
| Heelan et al., 2015 | Y | Y | Y | N | U | Y | N | Y | N | Y | U | 6 |
| Kridin et al., 2018 (b) | Y | N | Y | U | U | U | N | Y | N | Y | N | 4 |
| Kridin et al., 2018 (c) | Y | N | N | U | U | Y | N | Y | N | Y | N | 4 |
| Questions (Q): | | | | | | | | | | | | |
| Q1 Define the source of information (survey, record review) | | | | | | | | | | | | |
| Q2 List inclusion and exclusion criteria for exposed and unexposed subjects (cases and controls) or refer to previous publications | | | | | | | | | | | | |
| Q3 Indicate time period used for identifying patients | | | | | | | | | | | | |
| Q4 Indicate whether or not subjects were consecutive if not population-based | | | | | | | | | | | | |
| Q5 Indicate if evaluators of subjective components of study were masked to other aspects of the status of the participants | | | | | | | | | | | | |
| Q6 Describe any assessments undertaken for quality assurance purposes (e.g., test/retest of primary outcome measurements) | | | | | | | | | | | | |
| Q7 Explain any patient exclusions from analysis | | | | | | | | | | | | |
| Q8 Describe how confounding was assessed and/or controlled | | | | | | | | | | | | |
| Q9 If applicable, explain how missing data were handled in the analysis | | | | | | | | | | | | |
| Q10 Summarize patient response rates and completeness of data collection | | | | | | | | | | | | |
| Q11 Clarify what follow-up, if any, was expected and the percentage of patients for which incomplete data or follow-up was obtained | | | | | | | | | | | | |
| Y, Yes; N, No; U, Unclear | | | | | | | | | | | | |

| **1. Cohort studies** | | | | | | | | | |
| --- | --- | --- | --- | --- | --- | --- | --- | --- | --- |
| ***Studies*** | ***Selection*** | | | | ***Comparability*** | ***Outcome*** | | | ***Total score*** |
|  | ***Representativeness of the exposed cohort*** | ***Selection of the non-exposed cohort*** | ***Ascertainment of exposure*** | ***Demonstration that the outcome of interest was not present at the start of study*** | ***Comparability of cohorts on the basis of the design or analysis*** | ***Assessment of outcome*** | ***Follow-up long enough for outcomes to occur*** | ***Adequacy of follow-up of cohorts*** |  |
| Morioka et al., 1994 | 0.5 | 0 | 1 | 1 | 0 | 1 | 1 | 0 | 4.5 |
| Kyriakis et al., 1998 | 1 | 1 | 1 | 1 | 1 | 0 | 1 | 1 | 7 |
| Zeng et al., 2002 | 1 | 0 | 1 | 1 | 0.5 | 1 | 1 | 1 | 6.5 |
| Akarsu et al., 2017 | 1 | 1 | 1 | 1 | 0.5 | 1 | 1 | 1 | 7.5 |
| Kridin et al., 2018 (a) | 1 | 1 | 1 | 1 | 1 | 1 | 1 | 1 | 8 |

**Table S4. Quality assessment of the cohort and case-control studies using the Newcastle-Ottawa Scale**

| ***Possible source of heterogeneity*** | ***Number of studies*** | ***Coef (95% CI)*** | ***P-value*** |
| --- | --- | --- | --- |
| **Study design** | 16 | -0.008 [-0.032, 0.017] | 0.512 |
| Cohort study | 5 | -0.033 [-0.080, 0.014] | 0.154 |
| Cross-sectional study | 3 | -0.012[-0.059, 0.034] | 0.574 |
| Retrospective study | 8 | 0.023[-0.017, 0.062] | 0.236 |
| **Study Quality** | 16 | 0.017 [-0.012, 0.047] | 0.233 |
| Unknown | 8 | 0.023 [-0.017, 0.062] | 0.236 |
| Moderate quality (4~7) | 6 | -0.021 [-0.063, 0.022] | 0.311 |
| High quality (8~11) | 2 | -0.031 [-0.098,0.037] | 0.342 |
| **Region** | 16 | 0.016 [-0.010, 0.043] | 0.208 |
| Asia | 8 | 0.005 [-0.044, 0.053] | 0.837 |
| Europe | 4 | 0.031 [-0.026, 0.088] | 0.258 |
| North America | 4 | -0.013 [-0.059, 0.034] | 0.564 |
| **Study duration** | 16 | -0.017[-0.047, 0.014] | 0.258 |
| Less than 10 years | 8 | -0.036[-0.071, 0.000] | 0.049 |
| Between 10 to 20 years | 6 | 0.036[-0.002, 0.074] | 0.061 |
| More than 20 years | 2 | 0.011[-0.051, 0.073] | 0.714 |
| **Publish year** | 16 | 0.001[-0.021, 0.023] | 0.919 |
| Before 2000 | 3 | -0.007[-0.062, 0.049] | 0.803 |
| From 2000 to 2015 | 6 | 0.004[-0.038, 0.045] | 0.855 |
| After 2015 | 7 | -0.000[-0.041, 0.040] | 0.989 |

**Table S5. Meta-regression analysis of prevalence of tumor in patients with pemphigus**
